# Healthcare professionals’ views of palliative care for American war veterans with non-malignant respiratory disease living in a rural area: a qualitative study

**DOI:** 10.1186/s12904-019-0408-7

**Published:** 2019-02-27

**Authors:** Clare Mc Veigh, Joanne Reid, Paula Carvalho

**Affiliations:** 1 School of Nursing and Midwifery, Queen’s University Belfast, Belfast, UK; 2 Pulmonary and MICU, Boise VA Medical Centre, Boise, USA; 3 Division of Pulmonary and Critical Care Medicine, University of Washington, Seattle, USA

**Keywords:** Palliative care, Non-malignant respiratory disease, COPD, Interstitial lung disease, Bronchiectasis, Veterans, Healthcare professionals and rural

## Abstract

**Background:**

Chronic lung diseases, such as COPD, are a growing health concern within the veteran population. Palliative care programs have mainly focused on the needs of people with malignant disease in the past, however the majority of those worldwide needing palliative care have a non-malignant diagnosis. Additionally, palliative care provision can often be fragmented and varied dependent upon a patient’s geographical location. This study aimed to explore palliative care provision for veterans with non-malignant respiratory disease, and their family carers, living in a rural area of America.

**Methods:**

Qualitative study involving a convenience sample of 16 healthcare professionals from a large veteran hospital in Boise, Idaho. Data collection consisted of 5 focus groups which were transcribed verbatim and analysed using thematic analysis.

**Results:**

Healthcare professionals perceived that a lack of education regarding disease progression enhanced feelings of anxiety amongst veterans with NMRD, and their family carers. Additionally, the uncertain disease trajectory impeded referral to palliative and hospice services due to healthcare professionals own ambiguity regarding the veteran’s prognosis. A particular barrier also related to this particular patient population, was a perceived lack of ability to afford relevant services and a lack of local palliative service provision. Healthcare professionals expressed that a compounding factor to palliative care uptake was the perceptions held by the veteran population. Healthcare professionals expressed that alongside aligning palliative care with dying, veterans also viewed accepting palliative care as ‘surrendering’ to their disease. Findings indicated that telemedicine may be a beneficial platform to which palliative care can be provided to veterans with NMRD, and their family carers, in rural areas using a digital platform.

**Conclusion:**

Non-malignant respiratory disease is a life limiting condition commonly experienced within the veteran population. A new model of palliative care utilising a dynamic digital platform for this particular veteran population may provide an optimal way of providing efficient holistic care to areas with limited palliative services.

**Electronic supplementary material:**

The online version of this article (10.1186/s12904-019-0408-7) contains supplementary material, which is available to authorized users.

## Background

Globally, 210 million people have a diagnosis of chronic obstructive pulmonary disease (COPD) and, although the exact amount is not known, it is estimated that millions of others have another form of non-malignant respiratory disease (NMRD) [1]. Non- Malignant Respiratory Disease is an umbrella term that includes Interstitial Lung Disease (ILD), bronchiectasis and COPD [2]. Key international respiratory guidelines have highlighted the role of palliative care for patients with NMRD. The American Thoracic Society (ATS) emphasised the importance of palliative care being made available to patients with a respiratory illness and incorporated into their care from the point of diagnosis. The ATS also recommended that specialist palliative care should be involved when the patient’s needs go beyond the healthcare professional’s level of competency [3].

Statistics collated by the United States (US) Department of Veterans Affairs (VA) in 2016 highlighted that there were 20.4 million war veterans within America [4]. Chronic lung diseases, such as COPD, are a growing health concern within this veteran population. Veterans are three times more likely to develop COPD than the general population, and it is the fourth most prevalent condition amongst veterans [5]. The involvement of palliative care has been shown to reduce the number of emergency department visits, acute hospitalisations and admissions to Intensive Care Unit (ICU) experienced by veterans with a life limiting condition, including NMRD [6]. However the provision of palliative care to war veterans with a life-limiting illness presents unique challenges, in comparison to the general population. War veterans can experience multiple physical and psychological symptom complexities due to their exposure to war and combat situations [7, 8]. Amongst war veterans there is also often a culture of stoicism that can cause this patient cohort to under report their symptoms [9]. Evidence suggests that patients with NMRD and their caregivers do not receive the same standards of palliative care as patients with malignant respiratory disease [10, 11]. Geographical location can also impact the availability of local palliative service provision to patients with NMRD [12–14]. However, there is a dearth of evidence regarding the palliative care provision available for veterans with NMRD living in rural areas.

The US Department of VA is responsible for the Veterans Health Administration (VHA) which provides 1243 healthcare facilities to approximately 9 million American war veterans every year [15]. However there are large numbers of uninsured war veterans who do not utilise VHA services [16]. Additionally, American war veterans often live in sparsely populated areas that are located at a great distance from the nearest VA medical centre [17]. In comparison to urban areas, this population often experience suboptimal access to VHA services in rural communities [18]. Within Boise, Idaho, there is one VA Medical Centre that has a healthcare service area radius of approximately 160 miles with an estimated population of 100, 000 veterans [19]. Idaho averages 19 persons per square mile, compared to 87.4 persons per square mile for the US, and only six other states have a lower population density [20]. Idaho is a north-western state known for its mountainous landscapes and Boise is the capital. Boise VA provides primary and secondary care services to veterans with a range of acute and chronic complex conditions, who are often located in mountainous regions of the county.

### Aim

This study aimed to explore palliative care provision for veterans with non-malignant respiratory disease, and their family carers, living in a rural area of America.

## Methods

### Study design

This study had a qualitative design using focus groups (*n* = 5) with healthcare professionals (HCPs) involved in the care of veterans with a diagnosis of COPD, bronchiectasis or ILD. Due to the exploratory nature of this research it was important that the methodological approach taken aimed to explore the individual interpretations the participants associated with the phenomenon being investigated. Therefore, a broad interpretivist methodological approach was found to be the most suitable to answer the research question. Interpretivist researchers believe that by interacting with the world around them people attach their own meanings to a certain phenomenon, therefore interpretivist researchers attempt to access the meanings and values study participants apply to a phenomenon, in order to understand it [21].

### Recruitment and selection

**Fig. 1:**
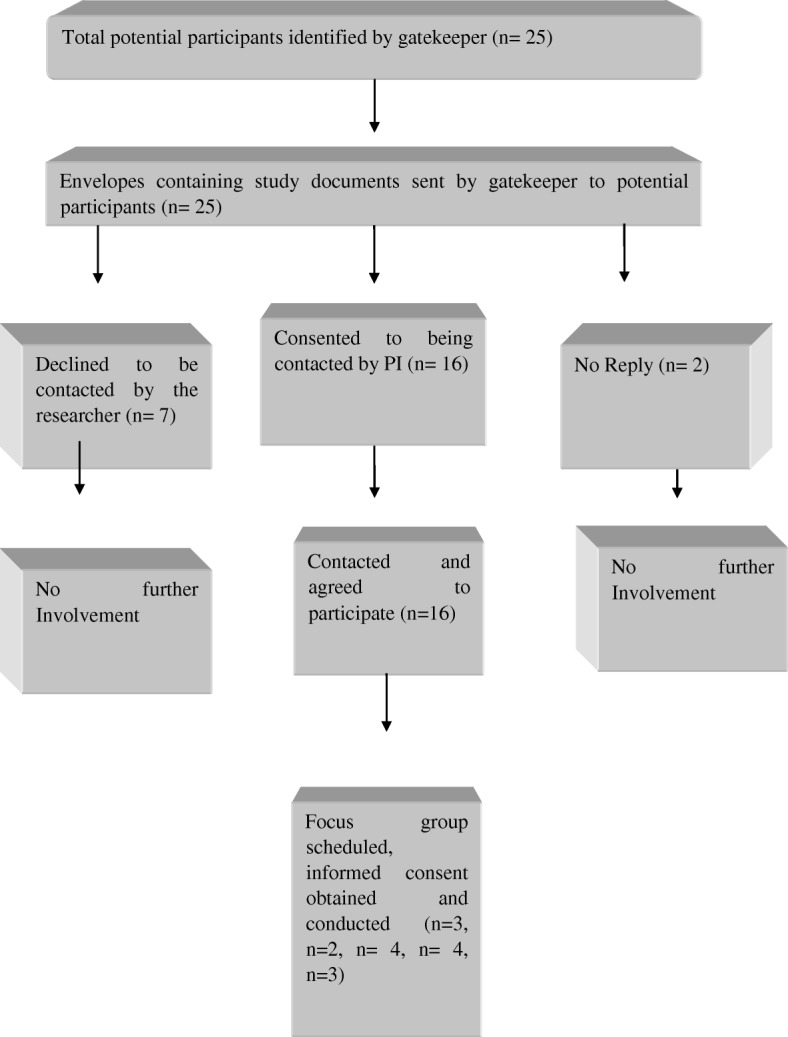
Flowchart of Recruitment and Retention

Healthcare professionals were recruited at a large veteran hospital in a rural area of America. Convenience sampling enabled choosing any individual whose experiences helped to achieve the aims of the study [22] and who met the eligibility criteria (Table 1). The recruitment process is outlined in Fig. 1. The medical director (gatekeeper) invited 25 HCPs to take part in the study and 16 agreed to be contacted about the study. Sixteen HCPs consented to take part in the focus groups in September 2014. Healthcare professionals were offered various date and time slots and invited to attend a focus group that was convenient for them.

**Table 1:** Eligibility criteria

Inclusion criteria	Exclusion criteria
Members of the multi-disciplinary team that are involved in the care of patients with non- malignant respiratory disease from the site involved in the study.	Are not employed by the site.
Have, or working towards, an appropriate professional qualification in their field of work.	
	Are not a healthcare professional involved in the care delivery to this client group.

### Data collection

Data was collected from healthcare professionals (Table 2) using 5 focus groups involving 2–4 healthcare professionals in each focus group. Focus groups may contain 2–4 participants if data is sufficient to demonstrate patterns when conducting thematic analysis [23]. The gatekeeper contacted healthcare professionals who consented to take part in the focus groups to arrange a suitable date and time for the focus groups to take place. Before the focus groups were commenced written informed consent was obtained from each participant. All focus groups were digitally recorded and transcribed verbatim. A focus group guide (Additional file 1) was developed by the project team using members’ experience of the topic area and previous research [21, 22]. Focus groups were conducted by the project team (PC and CMV) who had appropriate training on qualitative methods. To aid accurate recording of focus groups, a member of the project team fulfilled the role of note taker. Data collection was completed when it was noted by the lead author that no new themes or information were emerging from the data.

**Table 2:** Profile of participants

Participant number	Pseudonym	Job title
Focus Group 1		
1	MD1	Pulmonologist and Palliative Care Medical Physician
2	MD2	Pulmonologist
3	MD3	Pulmonologist
Focus Group 2		
4	RT1	Respiratory Therapist (Primary and Secondary Care Setting)
5	RT2	Respiratory Therapist (Primary and Secondary Care Setting)
Focus Group 3		
6	MS1	Chief resident
7	MS2	Internal Medicine Resident
8	MD4	Palliative Care Medical Physician
9	MS3	Medical Student
Focus Group 4		
10	RT3	Respiratory Therapist
11	RN1	Registered Nurse
12	RN2	Registered Nurse
13	RN3	Registered Nurse
Focus Group 5		
14	RN4	Registered Nurse
15	SW1	Social Worker
16	CM1	Case Manager (Registered Nurse for discharge planning)

### Data analysis

Data were analysed by adopting a thematic analysis framework described by [24]. To assist with managing the data the qualitative research software package NVivo [25] was used to provide the tools to support the management and categorisation of the qualitative data. Stage one of analysis was the assignment of descriptive themes to sections of the data in order to describe their meaning. Stage two encompassed grouping together descriptive themes to generate interpretative themes and highlight emerging patterns within the data. The third stage of the framework required the identification of a number of overarching themes developed by pulling together and linking all the interpretative themes that had been established. Each transcription was initially analysed by the lead author (CMV) and then reviewed by another member of the team (PC). All themes and transcripts were then discussed collaboratively by the project team (CMV, PC and JR) for verification and agreement of the themes generated [26].

## Ethical considerations

All procedures were reviewed and approved by the institutional review board (IRB) at the relevant institution. Verbal and written informed consent was obtained from each participant prior to commencement of the focus group. Any paper work produced throughout the focus group process is being held in a locked filing cabinet in a locked room, whilst any information stored on a computer is password protected. Pseudonyms have also been used to protect the identity of participants when verbatim quotations are used.

## Results

Analysis of the HCP focus groups identified two key overarching themes: 1) barriers to providing appropriate palliative care to veterans with NMRD; and 2) future direction of palliative care for veterans with NMRD. The overarching and interpretative themes are outlined in Table 3. Quotes are used as best exemplars from each theme and the anonymous participant codes for each quote are also included. Quotes have also been utilised to enhance rigour through thick description [27]. This involved the researcher providing explicit details of the experiences of the participants and moving beyond mere description of their accounts [22]. Holloway and Wheeler highlighted that this uncovers the meaning behind participants’ feelings and actions, and also allows for any reader of the study to decide if the research results can be applied across other settings.

**Table 3:** Overarching and interpretative themes

Overarching themes	Interpretative themes
1. Barriers to providing appropriate palliative care to veterans with NMRD	1(a) Lack of prognostic certainty 1(b) Misconceptions associated with palliative care and NMRD 1(c) Geographical location 1(d) Cultural background 1(e) Lack of palliative care services
2. Future Direction of Palliative Care for veterans with NMRD	2(a) Involvement of generalist and specialist palliative care 2(b) The role of telemedicine

### Theme 1: Barriers to providing appropriate palliative care to veterans with NMRD

Healthcare professionals linked an inability to provide appropriate palliative care to veterans with NMRD with difficulties in determining the patient’s prognosis:

*“The progression of interstitial lung disease can sometimes be very fast, people could have a normal life and then two months later be on 15 litres of oxygen at baseline…some of the times the barrier is just understanding [disease progression], which we don’t even understand everything about some of that stuff.”* (RT2)

*“If you see a patient like that [stage 4 lung cancer] in your clinic you’ve got a pretty good idea of how much time they have left….versus how many patients do we see with COPD that are oxygen saturations are 86% on room air and walking around doing things. The timeline just seems different and is less clear cut.”* (MS1)

Additionally, a lack of information regarding prognostication can cause feelings of distress amongst patients with NMRD and their family carers due a lack of awareness of the disease process:

*“I find that patients and families lack education and so they have a lot of anxiety, they panic, they want things done, especially for the patient who ... well, either the patient who is in some kind of distress and can’t cope or the family can’t cope watching them be in distress, because they're unaware of the disease process, and so anti-anxiety relief all around is needed with the education on this is what to expect.”* (RN1)

*“People [families] don't realise, the patient doesn't realise…that it [NMRD] can be a rapidly progressing disease and it can be something that end of life care or palliative care or comfort care needs to be addressed, but I think there’s not always that correlation based on the dynamic state of the disease.”* (RT2)

In America hospice care represents services that are available to patients nearing the end of life, and in order to receive hospice care physicians must state that a patient is in the last 6 months of their life. Some healthcare professionals perceive that the prognostication difficulties associated with NMRD can hinder the provision of hospice care to these patients:

*“I think my challenge is that to get palliative care started, it’s just a little difficult for non-cancer patients because of the life span. When we request hospice care you have to say a patient has six months to live, and that is very hard to judge for non-malignant, non-cancer respiratory conditions.”* (MD3)

*“We find that, at least in my practice I find a lot more confusion as to what these [NMRD] patients are actually going to be going through in terms of how long do they have, what's in it for them, what can we do, etc.”. (MD1)*

Participants acknowledged that HCPs still associate palliative care with cancer:

*“I think the other thing about (palliative care) services for malignant versus non- malignant is that if you ask a bunch of physicians or providers, nurses, whomever, should this person with stage four lung cancer be offered hospice, be offered palliative care? Almost uniformly people would say absolutely. If you say this person with COPD who is dyspnoeic at rest there would be a wide range of opinions about the timing of when palliative care, specialist palliative care, should be offered for the patient’s consideration.”* (MD4)

*“There’s (palliative care) resources for cancer patients, a phone number, and you’ve got somebody on the end of the line 24/7, but not so much for (non-malignant) lung disease. In my experience it's been very difficult to get services for patients with COPD, interstitial lung disease, different kinds of end stage pulmonary diseases, as compared with my patients, when we’re placing them, that have lung cancer, and the reason is because they don't necessarily send the specialised nurses nor the respiratory therapist to the home with as much frequency as they would to the patient that has cancer.”* (MD1)

Some HCPs conveyed that often veterans with NMRD are often reluctant to accept palliative care as they associated it with dying:

*“I think that is one thing that is confusing for patients, is that when you start talking about palliative care they think that it is hospice and they don't want to listen because they think it is now their time is limited and they have got end stage disease. So I think there is some confusion among the general public and resistance.”* (MD2)

*“Palliative care equates in their (veterans) mind with no treatment, essentially comfort care, which is not necessarily the case. We want their comforts but we also want to treat other things as they develop until the patient is eminently terminal and in their last few days. So sometimes patients and their families do not want palliative care involved.”* (MD1)

Healthcare professionals also perceived that veterans with NMRD often viewed palliative care as ‘surrendering’ to their illness:

*“I think that we have a very dynamic population (war veterans), whereas they are almost unwilling to admit that they would be giving up, and I think that's what palliative care is perceived as, as you know, you're just giving up. And so I think they kind of shy away from anything that would give them that feeling that they have surrendered, maybe. They don't like that, most of them.”* (RT2)

*“And men are very rightful and they don't want anybody to think that they're less of a man for wearing oxygen. I mean, they won’t wear it to the store because they don't want people to see them wearing their oxygen or they might not want to admit how short of breath they are and how debilitated they are because they want to be "strong men.”* (RT1)

Healthcare professionals indicated that veterans with NMRD, and their families, often receive fewer holistic services than those with a malignant condition:

*“We get so many patients [with NMRD] and families who get into trouble because we give them a bunch of machinery and then they get home with it and there's nobody really to help them with it, even a simple nebuliser many times, and I've had patients with cancer tied into this network.... For our COPDers (patients with COPD) and our interstitial lung disease patients...we don't have those resources easily available.”* (RN2)

Participants also perceived that veterans with NMRD are less likely to receive the appropriate level of palliative care services that they require if they live in rural areas:

*“You go into rural communities and it's the big goose egg and it becomes really problematic because these patients would like to go home and their families many times want them at home but then we can’t get the speciality RT or the speciality nurses, or maybe even an ER that's close. So I find that very challenging.”* (RN2)

*“In a rural area you will probably have a difficulty of getting better (palliative care) service compared to the urban area.” (MD3)*

Some participants perceived that a lack of HCPs working in the area of specialist palliative care in America created barriers when providing palliative care to veterans with NMRD:

*“There maybe are not enough palliative care specialists to take care of all the people who need palliative care.”* (MD4)

*“I think we still have gaps in specialist palliative care, in Idaho in particular, when sometimes people live in non-populous places, and so even if you could potentially get providers there to help, sometimes they're not terribly familiar with non-malignant respiratory illness.”* (MD4)

It was indicated that the gap in specialist palliative care physicians may be due to a lack of incentive for clinicians to specialise in palliative care:

*“The medicine pays for the physician to do procedures. If you do more procedures you get paid more, so people (physicians) are going to all the specialities where there are a lot of procedures. In hospice medicine there are not that many procedures….So unless the reimbursement is a little better and there is more incentive for people to go into (specialist) palliative care I think there may be a shortage and it will be hard to find a specialist (palliative care physician).”* (MD2)

Participants also reported that some veterans with NMRD may not be able to fully access palliative care services as they cannot afford them:

*“I don't think it's quite as easy to provide everything that they [veteran population with NMRD] need to maintain their quality of life, because we do liquid oxygen, we do whatever they need for oxygen, we're kind of in charge of that but out in the real world [outside the veteran hospital] it's run by what insurance wants to pay and what the patient ultimately can pay too.”* (RT1)

*“Finances will be a barrier, lack of finances, finances/resources, because then you (the veteran) can’t get the care that you need.”*(RN3)

### Theme 2: Future direction of palliative care for veterans with NMRD

Participants expressed that although generalist palliative care providers are not specifically trained in palliative care, it is part of their role:

*“I think palliative care should be something that everybody can do, regardless of their job, for every patient as they’re taking care of them.”* (MD4)

*“I feel that, yes, if they (generalist palliative care providers) know what they're doing, and also the palliative care hospital and Palliative Care Association of America has a very, very good website with all sorts of good treatment plans for every symptom that comes up. So if people can follow those and if they're doing the right thing I have no problem with a generalist doing it.”* (MD1)

Specialist palliative care involvement was perceived as necessary when a veteran with NMRD developed complex symptom needs:

*“There are good people that are here that I worked with that I think are very good at palliative care and are not board certified in palliative medicine (generalist palliative care providers) and I think they do a fabulous job and I think they involve (specialist) palliative care when they feel like they can’t help anymore.”* (MD4)

*“There is a programme in place here. It belongs to the medical called Packed ICU, and the Packed ICU is a team with nursing, social work, chaplain, psychology, pharmacy, people from medicine, generalists and specialists in palliative medicine, and this group, this team work together to develop a plan to help. Often it's to help palliate symptoms and then after this group has met and come up with some solutions there's usually a multi-faceted approach to treatment. For example, I had a patient that had non-malignant respiratory disease, and they got presented in one of these meetings, who also suffered from pretty severe anxiety. So we developed a treatment plan with psychology and pharmacy input to improve her medication regiment and then nursing checks in with this patient about one a month to do kind of a wellness check and make sure things are stable. I believe, my perception is that it had improved her quality of life.”* (MS1)

Participants also reported that having access to a multidisciplinary team that included generalist and specialist palliative care providers enhanced the palliative care experienced by this client group:

*“I dream of a system where the primary care docs (doctors in the community) get to phone a friend and the palliative care physician does the consultation with the primary care provider, such that they can continue to weave more palliative care into their general practice so that they're more comfortable with it.”*(MD4)

Telemedicine can be defined as the delivery of healthcare services and information through a digital platform [28]. Participants highlighted how the use of telemedicine is beneficial to healthcare professionals and patients, as palliative care can be delivered without the patient having to travel for long distances:

*“We now have a virtual clinic where we can sit down and call our patients and ask how is your breathing, how is your oxygen going, in a way that doesn't require them to load up into the car and drive several hours to get here, and I think that really helps because of the sort of philosophy of palliative care is to improve quality of life and make it so that people aren’t expending all their energy and getting short of breath and anxious having to come into physicians all the time, that that really helps people, that you can sort of reach out to them in their community and ask them how things are going and how well their medication is working, how their oxygen is doing and sort of help them improve their quality of life from afar.”* (MD4)

The role of family was also acknowledged by the majority of participants, and some placed great emphasis on the importance of involving the patient’s family in their care:

*“I think some of the facilitators (to palliative care), one thing I've noticed in our population, and especially with ILD or some of those things, is sort of greater family involvement. I think that when people come in with these diseases (NMRD) and they bring either their wife or children or everyone into an appointment with you and you can sort of communicate with them, everyone will take a different piece away and they might be able to better put that altogether*.” (MS2)

*“Having family members there as well (can improve services). Sometimes the patients are there and they are too busy focusing on breathing versus understanding what’s going on. He spouse or other family member needs to be there to really absorb the carer information.”* (RN2)

Participants also highlighted the importance of providing veterans with NMRD, and their families, with the appropriate knowledge to prepare for the life-limiting nature of the illness:

*“It seems that patients, and especially families, need a lot of education on what to expect and how to troubleshoot, like what to expect in the process with death and dying so they can be prepared.” (*RN3)

## Discussion

Findings concurred with previous research illuminating how the lack of prognostic certainty aligned with a NMRD diagnosis resulted in challenges identifying disease progression [13, 14, 27, 29]. Mc Veigh et al. [13] highlighted that HCPs particularly aligned prognostication difficulties in NMRD with a COPD or bronchiectasis diagnosis, due to perceptions that ILD displayed clearer prognostic indicators. However within the present findings HCPs associated prognostic uncertainty with all forms of NMRD, not just bronchiectasis and COPD. Furthermore, our findings illuminated that in America this impeded referral to hospice services within the primary care setting due to referral criteria indicating that patients must be within the last 6 months of life.

Healthcare professionals’ experiences additionally identified the impact of the NMRD trajectory on veterans and their family carers. Previous research has indicated that the uncertain disease trajectory associated with a NMRD can cause uncertainty amongst both patients and their family carers regarding how the disease will progress [29, 30]. Uncertainty related to disease progression amongst patients and carers is common in many non-malignant conditions such as heart failure [31], renal disease [32] and dementia [33]. The present findings highlighted perceptions that a lack of education regarding disease progression enhanced feelings of anxiety amongst veterans with NMRD, and their family carers. Healthcare professionals indicated the need for more education regarding NMRD and how to manage the condition, for both veterans and their family carers.

Internationally, patients with NMRD often have less access to specialist palliative care services than those with a malignant respiratory disease [11, 14, 34]. Present findings concurred with previous research due to participants perceptions that HCPs continue to predominately associate palliative care with a malignant diagnosis [11, 14]. In addition, our findings illuminated a compounding factor to palliative care uptake in that that the perceptions held by the veteran population often resulted in this client group’s reluctance to accept palliative care for NMRD. Healthcare professionals expressed that alongside aligning palliative care with dying, veterans also viewed accepting palliative care as ‘*surrendering’* to their disease.

A particular barrier related to veterans with NMRD living in America receiving optimal palliative care was a perceived lack of ability to afford relevant services. Chokshi and Sommes [35] previously argued that although there is a perception that all veterans’ healthcare needs are covered by the United States Department of Veteran Affairs Health System, many are not eligible or enrolled resulting in no coverage for their healthcare needs. Our findings have demonstrated that this is an issue that extends to palliative care, causing veterans who do not have appropriate healthcare cover to not receive the palliative service provision they require for their chronic respiratory condition.

Healthcare professionals’ accounts aligned with previous research regarding geographical location [12, 13, 36], highlighting that the generalist and specialist palliative care services in rural areas were limited in comparison to those available to veterans with NMRD living in urban areas. A novel perspective provided by the present findings however were HCPs’ perceptions that a lack of palliative care provision for veterans with NMRD in America, also resulted from a lack of physicians specialising in palliative care. A workforce taskforce for the American Academy of Hospice and Palliative Medicine previously illuminated a shortage of hospice and palliative medicine physicians in America that was potentially impeding on the delivery of specialist palliative care programs [37]. Our findings added to this view by suggesting that a lack of specialist palliative medicine physicians in America may be a result of a lack of financial incentive to specialise in this particular area.

Present findings provided HCPs’ perspectives on future models of care that may enhance the palliative care experienced by this particular veteran population. The accounts of HCPs concurred with previous research through acknowledging the important role specialist palliative care providers had in providing care to patients with NMRD who had complex symptom needs [14, 38–40]. However present findings additionally aligned with Higginson et al.’s [41] research, advocating the integration of early specialist palliative care for this client group. A potential model of care highlighted by present findings, that would be beneficial in providing appropriate holistic care to this particular client group, may involve the use of a telemedicine platform to deliver multi-professional generalist and specialist palliative care. Previous research has demonstrated how telemedicine can be effectively used to provide palliative consultations to critically ill patients [42].

These findings have implications for clinical practice, policy and research. Healthcare professionals should partake in education concerned with the holistic assessment and management of the needs of veterans with NMRD, and also the prognostic uncertainty associated with these conditions. This education also needs to develop their communication skills with regards discussing the trajectory associated with NMRD with veterans and their families. Optimal information giving may also contribute to an enhanced understanding of the role of palliative care amongst war veterans, which could aid in dispelling misconceptions. Future education delivery models for HCPs involved in the care of veterans with NMRD may employ the use of the Extension for Community Healthcare Outcomes (ECHO) model [43–45], which utilises digital education platforms to enhance speciality training and support for clinicians delivering complex care to populations in remote areas.

To reduce inequalities in the provision of palliative care provided to veterans with NMRD related to geographical location, service development measures must be put in place [46], such as those identified within the present study. Telemedicine may be an effective platform that could be utilised within clinical practice to deliver palliative care to war veterans living in areas with limited local palliative service provision. Future research could adopt the Medical Research Council (MRC) framework for complex interventions [47] in order to investigate the use of a telemedicine model of palliative care derived from the findings of this study. This would initially involve conducting a feasibility study with respiratory, generalist palliative care and specialist palliative care teams to explore the practicalities of implementing the proposed model for veterans with ILD, COPD and bronchiectasis.

### Limitations

Findings only represented the perspectives of HCPs and not the patient’s own perspective. This study may have benefitted from gaining the perspectives of patients and family carers to gain further insight into their experiences of palliative care for veterans with NMRD. Additionally, this study only involved one veteran hospital and may have been enhanced through using multiple sites.

## Conclusion

Non-malignant respiratory disease is a life limiting condition that is more commonly experienced within the veteran population, than the general population. This study has suggested that HCPs perceive there to be significant challenges in providing palliative care to this population due to misconceptions held by HCPs and veterans, and a lack of palliative service provision. A lack of local palliative service provision can be particularly challenging for veterans with NMRD, and their family carers, living in areas of great rurality. A new model of palliative care utilising a dynamic digital platform for this particular veteran population may provide an optimal way of providing efficient holistic care to areas with limited palliative services.

## Additional file

Additional file 1: Focus Group Question Guide. (DOCX 15 kb)

## Abbreviations

ATS: American Thoracic Society
COPD: Chronic Obstructive Pulmonary Disorder
HCP: Healthcare Professional
ICU: Intensive Care Unit
ILD: Interstitial Lung Disease
IRB: Institutional Review Board
NMRD: Non-Malignant Respiratory Disease
