# Supplementary material for: Healthcare professionals’ views of palliative care for American war veterans with non-malignant respiratory disease living in a rural area: a qualitative study
Source: BMC Palliat Care. 2019 Feb 27;18:22. doi: 10.1186/s12904-019-0408-7 (PMC6394001; doi:10.1186/s12904-019-0408-7)
Supplement: Supplementary file 1 — Focus Group Question Guide. (DOCX 15 kb) [file 12904_2019_408_MOESM1_ESM.docx]

**Supplement 1**

**Focus Group Question Guide**

**Introduction**

- Welcome everyone
- Introduce myself
- House Keeping
- Reminder about the study: what it is about; why I am doing it
- Talk through key points
- voluntary
- confidentiality and digital recording device
- length of focus group
- group discussion- everyone join in
- no right or wrong answers, ok to disagree with others, want to hear everyone’s opinion
- respect others views, no interrupting and no ‘side’ conversations
- explain role of note taker in the background
- any questions?
- **Definition of palliative care=** The WHO (2002) definition is,

“An approach that improves the quality of life of patients and their families facing the problems associated with life-threatening illness, through the prevention and relief of suffering by means of early identification and impeccable assessment and treatment of pain and other problems, physical, psychosocial and spiritual.”

- **Definition of specialist palliative care (SPC) =** The palliative care provided by healthcare professionals (HCP) that specialize in the area of palliative care.
- **Definition of generalist palliative care (GPC) =** Palliative care delivered by HCPs who are not classified as specialist palliative care providers.

**Warm Up**

- Tell me a bit about yourself
- Name, professional role, area they work in, how long they have worked there……

**Topic Guide**

1. **Aim: To explore what palliative health service provision is currently available for patients with non-malignant respiratory disease (NMRD) and their caregivers.**

To start, could anyone tell me about the palliative health care provision that is currently available for people with non- malignant respiratory disease and their carers, both specialist and generalist?

- What do you feel would make for a high standard of pall care post discharge for these patients and carers?
- Can you tell me how well GPC and SPC services are organized and resourced both in the hospital and community setting?
- Can you tell me how effective are the lines of communication between the HCPs involved? (Especially between GPC and SPC)
- Can you tell me how well GPC and SPC services are accessed by this client group both in the hospital and community setting? (How aware are they of all available services?)
- Would anyone agree/ disagree with this?
- Would anyone have any other perspective?
- How do you perceive patients and their carers understand of the meaning of palliative care?
- Would anyone agree/ disagree with this?
- Would anyone have any other perspective?
- What do you perceive as good palliative care?
- What are the barriers and facilitators of good palliative care?
- Any suggestions for improvement?
- How do you feel good generalist palliative care compares with specialist palliative care?
- How do you perceive the GPC and SPC services available to patients with a diagnosis of either bronchiectasis or interstitial lung disease (ILD) and their carers, compares to the services available to those with a diagnosis of COPD?
- How do you perceive the GPC and SPC services available to NMRD patients and their carers in rural areas compares to the services available to those in urban areas?
- Can you tell me about any Models of Palliative Care that are used at present to guide the provision of palliative care?
- How do you perceive the role of the Healthcare Support Worker in delivering palliative care to patients at home? (Importance, what they do, standard, do they need training??)

1. **Aim: to find out if the HCP’s feel they communicate well enough with patients and carers and if they meet the information needs of patients and carers.**

Can you tell me how you perceive how GPC and SPC providers communicate with NMRD patients and their carers?

- Can you tell me your perceptions about how the information needs of carers and patients are met?
- Would anyone agree/ disagree with this?
- Would anyone have any other perspective?
- Can you tell me about the barriers and facilitators in relation to communicating with patients and carers?
- Any suggestions for improvement?
- Can you tell me how you feel about how effective discussions around end-of-life and prognosis with patients and carers are?
- What are the barriers involved in having these conversations?
- Any suggestions for improvement?
- How effective do you feel HCPs are at taking into consideration the wishes of the patient and their family?
- Would anyone agree/ disagree with this?
- Would anyone have any other perspective?
- How effective do you think the bereavement care is that is provided to the bereaved caregivers involved with this client group?
- Would anyone agree/ disagree with this?
- Would anyone have any other perspective?

**Anything else you would like to add to the discussion which we have not covered?**

**Thank you.**
